# Are Phenotypic Baseline Characteristics Related to Abolition of AHR After Benralizumab and Dupilumab?

**DOI:** 10.1111/cea.70279

**Published:** 2026-03-10

**Authors:** Philipp Suter, Robert Greig, Rory Chan, Brian J. Lipworth

**Affiliations:** ^1^ Scottish Centre for Respiratory Research University of Dundee UK

## Abstract

- Lower baseline FEV1 and PEF predicted persistence of mannitol airway hyperresponsiveness to biologics.
- Higher baseline eosinophils predicted abolition of mannitol airway hyperresponsiveness to biologics.

**Abbreviations**
ACQ: Asthma Control Questionnaire
AHR: airway hyperresponsiveness
ASM: airway smooth muscle
BMI: body mass index
ERS: European Respiratory Society
FeNO: fractional exhaled nitric oxide
FEV_1_
: forced expiratory volume in 1 s
FVC: forced vital capacity
PD10: provocative dose causing a 10% fall in FEV_1_
PEF: peak expiratory flow
SEM: standard error of the mean
T2: type 2

To the Editor,

Airway hyperresponsiveness (AHR) is a key feature of uncontrolled severe asthma, representing a valuable tool for assessing disease activity, and is associated with airway structural changes and exacerbation risk [1].

AHR can be assessed using either direct or indirect challenge tests. Direct challenge tests, such as those using methacholine or histamine, induce bronchoconstriction by acting directly on airway smooth muscle (ASM) receptors. In contrast, indirect challenge tests provoke bronchoconstriction via osmotic and inflammatory mechanisms. Chemical stimuli, such as mannitol, act via mast cells and eosinophils, in turn, triggering the release of inflammatory mediators such as histamine, leukotriene D4 and prostaglandins. Consequently, ASM constriction is a sequential process and more physiological [2].

The arrival of biologic therapies has revolutionised the management of uncontrolled severe asthma. Notably, several studies have demonstrated that biologic therapies can significantly reduce AHR in patients with severe asthma [3, 4].

Type 2 biomarkers and lung function parameters have been identified to predict a favourable clinical response to biologic therapies. However, it remains difficult to predict which individuals may achieve abolition of AHR in response to biologics. To our knowledge no study has assessed baseline characteristics as a prognostic factor for abolition of AHR with biologics in severe uncontrolled asthma. Abolition of AHR with biologics has been suggested as a treatable trait and be included in the definition of complete remission [5].

We performed a post hoc analysis of biologic‐naïve patients with severe uncontrolled type 2 (T2) high asthma from two prospective clinical trials (EudraCT 2019‐003763‐22 and EudraCT 2021‐005593‐25) with the aim of identifying baseline characteristics which predict abolition of mannitol AHR in response to either benralizumab or dupilumab [6, 7]. Both trials were conducted using identical inclusion criteria, mannitol challenge methodology and follow‐up time. The studies were pooled to increase statistical power for identifying baseline determinants of AHR abolition at a patient level rather than to compare drug efficacy.

All patients included underwent AHR assessment using mannitol challenge at baseline and after 3 months of treatment. We compared baseline characteristics between patients with persistent AHR (AHR^+^), defined as the provocative dose causing a 10% fall (PD10) in FEV_1_ of < 635 mg, and those demonstrating abolition of AHR (AHR^−^) as mannitol PD_10_ FEV_1_ ≥ 635 mg. Spirometry (Micromedical, Chatham, UK) was performed in triplicate in accordance with European Respiratory Society (ERS) guidelines. Fractional exhaled nitric oxide (FeNO) was measured using NIOX Vero (NIOX, Oxford, UK) in accordance with the ERS guidelines.

Independent *t*‐tests were performed using a two‐tailed significance level (alpha) of 5%. Nominal *p* values are reported as < 0.05, < 0.01, or < 0.001. Since baseline eosinophil levels were not normally distributed, their values were log transformed for analysis. SPSS version 30 (IBM Corp, Armonk, NY, USA) was used to conduct statistical analyses.

The cohort consisted of 42 patients, 48% female, mean age 54.4 years and BMI 29.9 kg/m^2^. All patients were treated with inhaled corticosteroids and a mean (SEM) beclomethasone dipropionate equivalent dose of 1565.0 μg (78.4 μg). Baseline mean (SEM) values were: FEV_1_ 2.31 L (0.10); FEV_1_% predicted 80.41% (2.55); FEV_1_/FVC ratio 65.36% (1.35); peak expiratory flow (PEF) 384 L/min (15); FeNO 46.4 ppb (5.1), blood eosinophils 479 cells/μL (42), asthma control questionnaire (ACQ‐6) 2.46 (0.13), mannitol PD10 164.35 mg (23.84). AHR was abolished in 14 of 22 patients treated with dupilumab and in 4 of 20 patients treated with benralizumab. There were no significant differences between the benralizumab and dupilumab groups in regard to age, body mass index, beclomethasone dipropionate equivalent dose and smoking history (none were current smokers). In the dupilumab group (96%) significantly more patients had chronic rhinosinusitis with nasal polyposis compared to the benralizumab group (38%).

Comparing AHR^−^ versus AHR^+^ groups, the former patients demonstrated higher baseline blood eosinophils amounting to a geometric mean fold difference 1.39 (95% CI 1.02, 1.90), *p* < 0.05, a higher FEV_1_: mean difference of 410 mL (95% CI 10, 810), *p* < 0.05, and a higher PEF: mean difference of 78 L/min (95% CI 19, 137), *p* < 0.01 (Figure 1A–C). No significant differences comparing AHR^−^ versus AHR^+^ patients were observed in FeNO: mean difference: 0.53 ppb (95% CI −21.73, 20.67), or ACQ‐6: mean difference 0.26 (95% CI −0.25, 0.78) (Figure 1D,E). Moreover, baseline geometric mean PD10 was higher in the AHR^−^ versus AHR^+^ (geometric mean difference 2.42 (95% CI 1.09, 5.34), *p* < 0.05). The association between baseline characteristics and AHR abolition remained directionally consistent when analysing separately for benralizumab and dupilumab, respectively.

**FIGURE 1:**
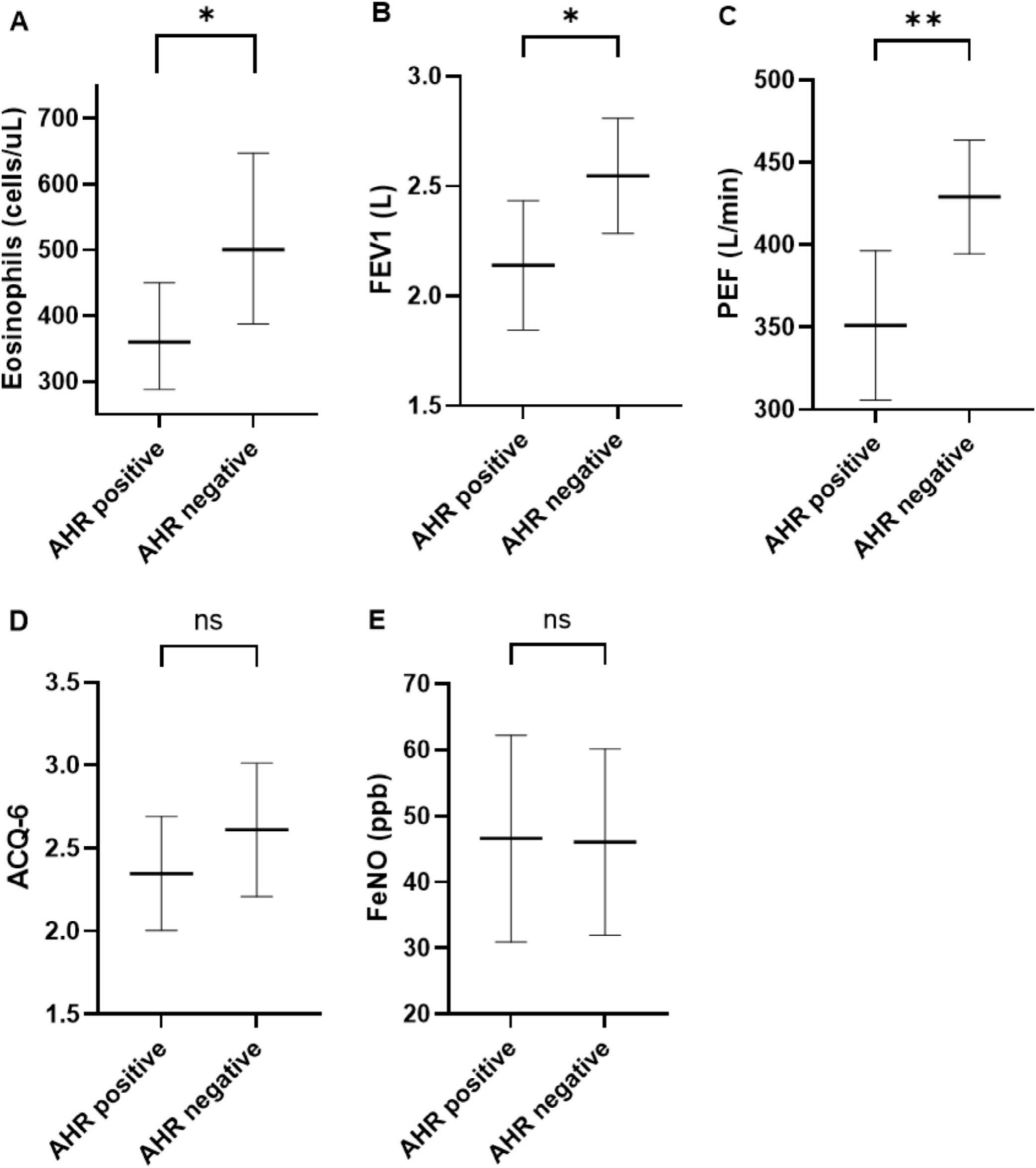
Comparison of baseline characteristics between AHR^+^ (*N* = 24, dupilumab *n* = 8, benralizumab *n* = 16) and AHR^−^ (*N* = 18; dupilumab *n* = 14, benralizumab *n* = 4) patients after biologic treatment. Data are depicted as arithmetic means and 95% CI except for eosinophils as geometric means. *p < 0.05, **p < 0.01. ACQ‐6, asthma control questionnaire; AHR, airway hyperresponsiveness; FeNO, fraction of exhaled nitric oxide; FEV1, forced expiratory volume in 1 s; FVC, forced vital capacity; PEF, peak expiratory flow.

Our results showed that patients who had persistent mannitol‐defined AHR after benralizumab or dupilumab treatment exhibited lower blood eosinophils and worse lung function at baseline. Current evidence indicates that higher levels of baseline blood eosinophils predict a better clinical response to benralizumab and dupilumab, as reflected by effects on exacerbations, symptom control and quality of life [8]. Here we also demonstrate that this predictive value of eosinophils extends to AHR in that patients with higher eosinophils were more likely to achieve abolition of AHR. Notably, there was an apparent disconnect in baseline T2 biomarkers, in terms of blood eosinophils reflecting IL5, but not FeNO reflecting IL13, being able to discriminate between patients who had persistent or abolished AHR in response to benralizumab or dupilumab. This is perhaps counterintuitive as one might expect that higher FeNO levels at baseline would be associated with abolished AHR in response to dupilumab given that there is high expression of IL13 on ASM [9].

Altered baseline airway geometry assessed by FEV_1_ and PEF may reflect epithelial damage along with basement membrane and airway wall thickening. Consequentially in line with current evidence, reduced airway calibre accompanied by worse lung function may in turn contribute to persistent AHR [1]. Unsurprisingly, given the higher baseline FEV1 value in the AHR^−^ group, the same patients also had a higher baseline PD10 due to effects of airway geometry.

Hence, baseline eosinophils and lung function may predict effects of benralizumab and dupilumab on mannitol AHR and help in achieving a more individualised approach to biologic treatment, perhaps including earlier initiation to obviate persistent AHR. However, in the present post hoc analysis, limited sample size and short period of follow up are worth mentioning as potential limitations. We also appreciate that proportionately more patients treated with dupilumab than benralizumab achieved abolition of AHR. In this regard, one might have expected the presence of higher baseline eosinophils to exert a greater influence on the AHR response to benralizumab than dupilumab. Prospective long‐term studies are required to further identify which markers may predict abolition of AHR with biologics.

## Author Contributions

Philipp Suter: data curation, formal analysis, investigation, writing – original draft. Robert Greig: formal analysis, writing – review and editing. Rory Chan: writing – review and editing. Brian J. Lipworth: conceptualization, formal analysis, writing – review and editing.

## Funding

The authors have nothing to report.

## Conflicts of Interest

Philipp Suter reports a relationship with AstraZeneca UK Limited that includes: speaking and lecture fees. Philipp Suter reports a relationship with GSK that includes: speaking and lecture fees. Philipp Suter reports a relationship with Lung League Fribourg that includes: funding grants and lecture fees. Philipp Suter reports a relationship with Swiss Lung Foundation that includes: funding grants. Robert Greig reports a relationship with AstraZeneca UK Limited that includes: speaking and lecture fees and travel reimbursement. Rory Chan reports a relationship with Asthma and Lung UK that includes: funding grants. Rory Chan reports a relationship with Chiesi Pharmaceutical that includes: funding grants, speaking and lecture fees and travel reimbursement. Rory Chan reports a relationship with AstraZeneca UK Limited that includes: board membership, consulting or advisory, funding grants, speaking and lecture fees and travel reimbursement. Rory Chan reports a relationship with GSK that includes: funding grants. Rory Chan reports a relationship with Vitalograph UK Ltd. that includes: board membership, speaking and lecture fees and travel reimbursement. Rory Chan reports a relationship with Thorasys that includes: speaking and lecture fees. Rory Chan reports a relationship with Sanofi that includes: travel reimbursement. Rory Chan reports a relationship with NIOX Group Plc that includes: travel reimbursement. Brian J. Lipworth reports a relationship with AstraZeneca UK Limited that includes: board membership, funding grants and speaking and lecture fees. Brian J. Lipworth reports a relationship with Sanofi that includes: board membership, speaking and lecture fees and travel reimbursement. Brian J. Lipworth reports a relationship with Chiesi Pharmaceutical that includes: consulting or advisory, funding grants, speaking and lecture fees and travel reimbursement. Brian J. Lipworth reports a relationship with Lupin Pharmaceuticals Inc. that includes: consulting or advisory. Brian J. Lipworth reports a relationship with Glenmark Pharmaceuticals Limited that includes: consulting or advisory and speaking and lecture fees. Brian J. Lipworth reports a relationship with Sandoz Inc. that includes: consulting or advisory. Brian J. Lipworth reports a relationship with Vitalograph Ltd. that includes: funding grants. Brian J. Lipworth reports a relationship with Thorasys that includes: funding grants. The son of Dr. Brian J. Lipworth is presently an employee of AstraZeneca.

## Data Availability

The data that support the findings of this study are available from the corresponding author upon reasonable request.
